# Hotter deserts and the impending challenges for the Spiny-tailed Lizard in India

**DOI:** 10.1242/bio.060150

**Published:** 2024-03-02

**Authors:** Avichal Tatu, Sutirtha Dutta, Maria Thaker

**Affiliations:** ^1^ Wildlife Institute of India, Dehradun, Uttarakhand, 248001, India; ^2^ Centre for Ecological Sciences, Indian Institute of Science, Bangalore, Karnataka, 560012, India; ^3^ School of Biosciences, University of Melbourne, Parkville, Victoria, 3052, Australia

**Keywords:** Climate change, Ectotherm, Desert, Foraging, Spiny-tailed lizard, Microhabitat, Thermal performance, Thermal ecology

## Abstract

Ectotherms are particularly vulnerable to climate change, especially those living in extreme areas, such as deserts, where species are already thermally constrained. Using the vulnerable herbivorous lizard *Saara hardwickii* as a model system, we used a multi-pronged approach to understand the thermal ecology of a desert agamid and potential impacts of rising temperatures. Our data included field-based measures of operative temperatures, body temperatures, and activity, as well as lab-based measures of thermal limits, preferences, and sprint speed. As expected, the temperature dependence of locomotor performance and foraging activity were different, and in the worst-case global warming scenario (SSP5-8.5), potential sprint speed may decrease by up to 14.5% and foraging activity may decrease by up to 43.5% by 2099. Burrows are essential thermal refuges, and global warming projections suggest that *S. hardwickii* may be restricted to burrows for up to 9 h per day by 2099, which would greatly limit critical activities, like foraging and seeking mating opportunities. Overall, we show that key information on thermal ecology, including temperature-sensitive behaviours in the wild, is necessary to understand the multiple ways in which increasing temperatures may influence ectothermic vertebrates, especially for species like *S. hardwickii* that are already vulnerable to environmental change.

## Teaser

**Summary:** Observations of foraging activity in the wild play an important role in predicting the impacts of climate change in a herbivorous desert lizard, supplementing lab-based measurements of thermal variables.

## INTRODUCTION

Climate change is one of the major drivers of biodiversity change in the current century (Sala et al., 2000). With climatic projections forecasting an increase in global temperatures of up to 4°C by the end of this century (IPCC, 2019), nearly one million species of plants and animals are estimated to face the risk of extinction (IPBES, 2019)*.* Ectotherms are particularly susceptible to the impacts of climate change since performance and fitness are highly dependent on environmental temperatures (Abram et al., 2017; Angilletta, 2009). Many models use physiologically based thermal tolerance limits as criteria to generate broad-scale predictions of ectotherm extinction probabilities under global warming scenarios (Huey et al., 2010; Sinervo et al., 2010). Ectotherms, however, are not completely vulnerable to environmental conditions. Behavioural thermoregulation strategies, such as seeking refuges or adjusting activity windows, can maintain body temperature (T_b_) within preferred ranges, even in sub-optimal conditions (Huey et al., 2003; Logan et al., 2019). Predictive models that exclude coping strategies, such as the use of thermal refugia, the thermal variability of different microhabitats, the capacity to thermoregulate, or activity patterns, may over- or under-estimate the potential effects of climate warming on species (Briscoe et al., 2016; Gunderson and Leal, 2015; Moore et al., 2018). Because climate change is predicted to impact behaviour (Abram et al., 2017) through physiology (e.g. Burraco et al., 2020; Kingsolver et al., 2013), approaches to understand the impacts of climate change requires measurements of the thermal environment that species experience as well as their responsive strategies (Gates, 2012; Gunderson and Leal, 2016).

Lizards are ideal model organisms to study thermal biology and the impacts of climate warming on ectotherms (Pianka, 1986). As with most ectotherms, the relationship between temperature and critical functional processes, such as locomotion and growth, is typically characterized by a non-linear and asymmetrical curve, peaking at an optimum temperature where performance is at its highest level (T_opt_). To maintain body temperatures within their preferred range around T_opt_, lizards use a wide range of thermoregulatory strategies, which include metachrosis, postural changes, altering activity times, and shuttling between shaded and open microhabitats (Angilletta, 2009). To prevent overheating when temperatures exceed thermal limits, lizards that are behavioural thermoregulators seek thermal refuge. Using refugia, however, has costs as animals usually cannot forage, mate or defend territories during that time. Many have predicted that the temperature increases due to global warming will increase the hours in which lizards are restricted in their refugia. Using hours of restriction (h_r_) as a measure, Sinervo et al. (2010) predicted that by 2080, local extinctions of lizards may reach 39%, and total species extinction may reach 20%. Predictive models based on h_r_ that are calculated using thermal tolerance limits (Sinervo et al., 2010), however, might underestimate the activity budgets of lizards because they do not take into account variation in other behaviours that might also be influenced by temperature (Gunderson and Leal, 2016; Kearney, 2013).

Lizards surviving in areas with extreme climates, such as deserts, are even more vulnerable to the impacts of climate change since environmental temperatures often exceed their thermal thresholds (Huey et al., 2009; Ivey et al., 2020; Sunday et al., 2014). Hardwicke's spiny-tailed lizard, *Saara hardwickii* is a diurnal herbivorous lizard dwelling in arid and semi-arid areas of India, Pakistan, and Afghanistan. (Das et al., 2013; Dutta and Jhala, 2007; Khan and Mahmood, 2004). This species is under the vulnerable category of the IUCN (Vyas et al., 2022). In addition to severe poaching and habitat loss due to cropland expansion (Dutta and Jhala, 2014), *S. hardwickii* also faces the thermal challenges of living in a desert ecosystem. The Thar desert of Jaisalmer district in Rajasthan, India, where densities of *S. hardwickii* are high (Ramesh and Ishwar, 2008), is one of the hottest areas in India (Booth et al., 2014). Air temperatures can reach up to 50°C in this region. It is in this landscape that we studied the thermal ecology of this agamid and its vulnerability to climate change. From systematic field measures, we obtained data on body temperature (T_b_), operative temperatures (T_e_), and activity pattern, which enabled us to quantify the effectiveness of thermoregulation and temperature-dependent activity. These data from the wild also enabled us to determine the voluntary thermal maxima (VT_max_) or average daily maximum T_b_, an ecologically-relevant measure of a thermal threshold that indicates the temperature at which individuals of the species seek thermal refuge in burrows (Camacho and Rusch, 2017; Ivey et al., 2020). Using wild-caught lizards brought into a controlled laboratory condition, we measured the critical thermal maxima (CT_max_), critical thermal minima (CT_min_), preferred temperature (T_set_), and thermal performance curve of locomotion. This is the first study of thermal ecology for any lizard in India. With these data, we explore how temperature influences the physiology and behaviour of a desert vertebrate, and evaluate how projections of temperature increases may impact h_r_, locomotor performance, and foraging activity. With an integrated approach that incorporates measures of behaviour in the wild and thermal limits in the laboratory, these data provide a multivariate understanding of the vulnerability of this threatened species.

## RESULTS

### Operative temperatures (T_e_) in the wild

There was a significant effect of both microhabitat (*F(2,15483)*=1087.4, *P*<0.001) and month (*F(1,15483)=*909.2, *P*<0.001) on T_e_, with no significant interaction between the two variables (*F(2,15483)=*2.1, *P*=0.14). Above ground environmental temperatures in the open (T_e_ open; *β=*36.4, *T=*310.6, *P*<0.001) were significantly higher than temperatures in the burrow (T_e_ burrow; *β=*31.8, *T*=356.4, *P*<0.001). T_e_ open at the two study sites were not significantly different (*F(1,1266)*=0.1, *P*=0.75). Mean environmental temperature (T_e_ open) in March was 33.3°C±0.2°C s.e. compared to 36.4°C±0.2°C s.e. in April and 38.9°C±0.2°C s.e. in May.

### Field active body temperature (T_b_) and effectiveness of thermoregulation (E)

**Fig. 1.**
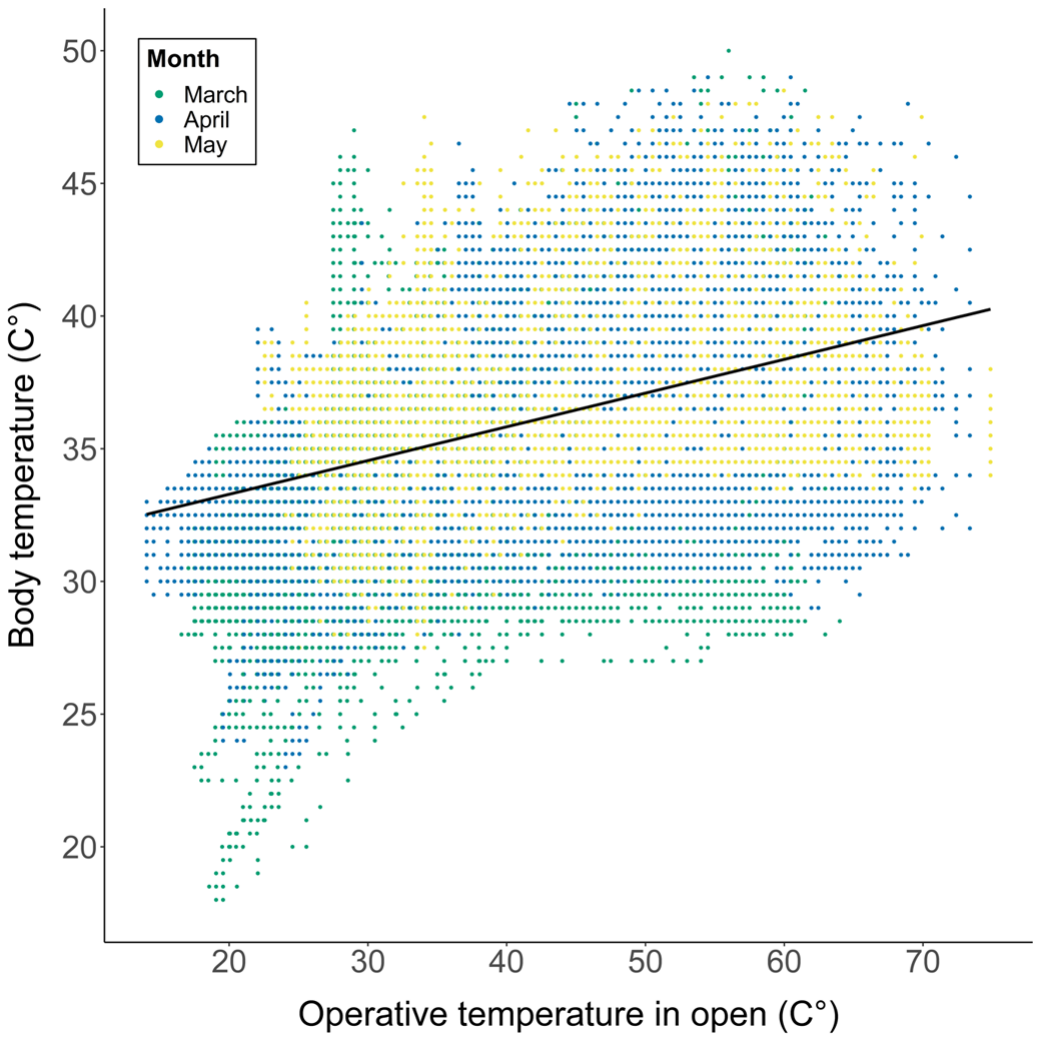
**Body temperatures of *Saara hardwickii* (*n*=19) as a function of operative temperatures (T_e_) in open environments during the months of March (green), April (blue) and May (yellow).** Line shows a linear regression slope.

A total of 65,952 field active body temperature (T_b_) data points from 19 individuals were obtained. In March, the mean field T_b_ was 33.6°C±0.03°C s.e. compared to 35.2°C±0.01°C s.e. in April and 36.5°C±0.01 May. The effect of T_e_ open on field T_b_ was significant and positive (*β=*0.12, *F(1,65934)*=18,529, *P*<0.001; Fig. 1). There was also a significant but weak effect of BCI on T_b_ (*β=*−0.01, *F(1,15)*=4.62, *P=*0.048), but no significant effect of sex (*β=*0.65, *F(1,15)*=2.52, *P=*0.13) and site (*β=*0.58, *F(1,15)*=2.05, *P*=0.17). There was a significant random effect of 'individual' (G^2=^5314.6, *P*<0.001) on T_b_. Based on the field measures of T_b_, we found that VT_max_ of *S. hardwickii* was 46.3°C±0.3°C s.e.

The mean thermoregulatory accuracy (*d_b_*) for all T_b_ data was 3.45±0.01 (ranging from 0 to 20.2), and the mean thermal quality (*d_e_*) of all microhabitats combined was 7.54±0.03 (ranging from 0 to 34.9). Overall effectiveness of thermoregulation (*E*) of *S. hardwickii* was 0.54, suggesting that this species is a moderately precise thermoregulator. We found that extent of thermoregulation was highest in May (*E*=0.6) followed by April (*E*=0.56), and March (*E*=0.44) indicating a higher effectiveness of thermoregulation during the hotter months.

### Temperature-dependent activity

The results of TBAE showed substantial agreement with our test data (82.7%; K_=_0.6; *P*<0.001). Using TBAE, the proportion of activity by lizards was found to be 6% higher in March compared to April and May. Total proportion of activity was the same during April and May, with a greater proportion of lizards being active in the mornings and evenings compared to the afternoon or night during both those months (see Fig. S4).

**Fig. 2.**
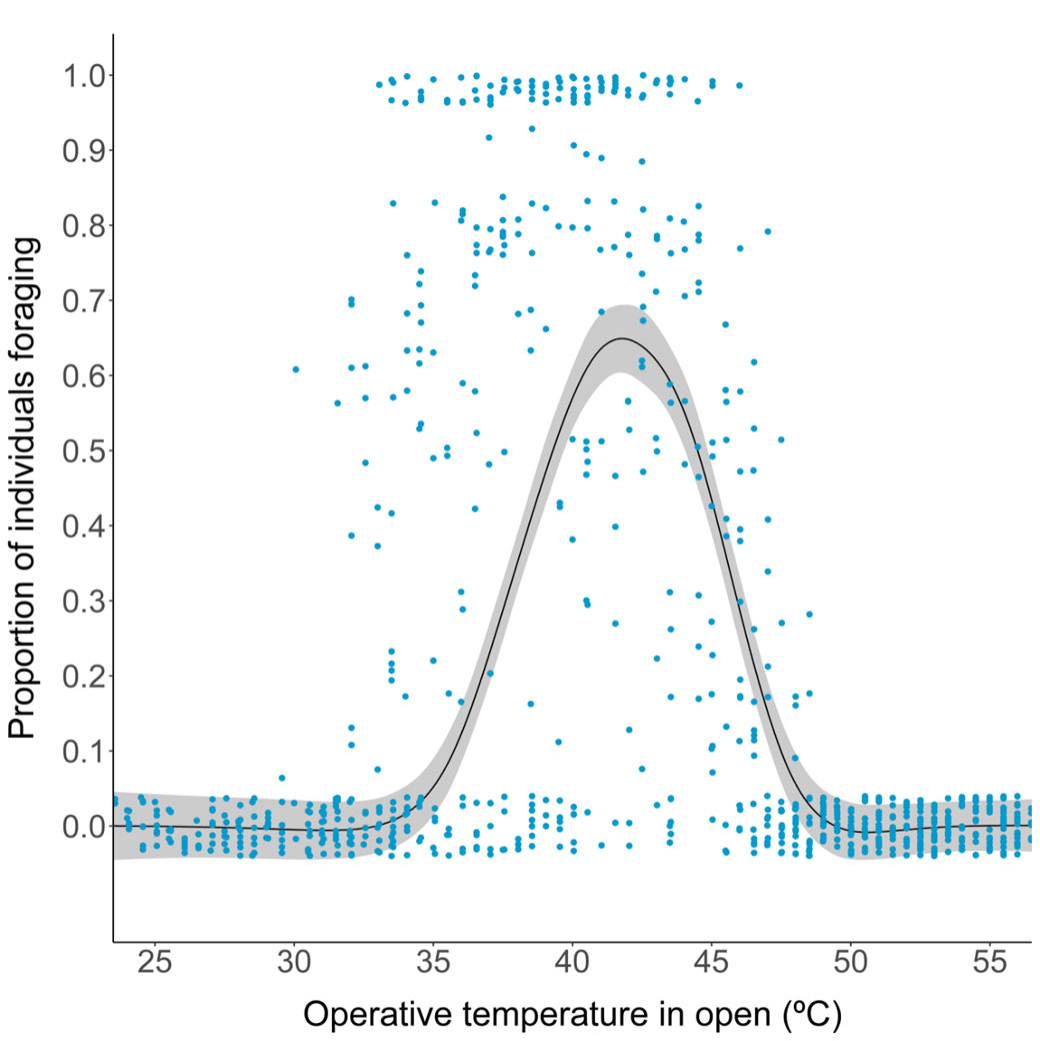
**Temperature-dependence of foraging activity in *Saara hardwickii*, measured as the proportion of individuals recorded foraging during field scans (*n*=1127 scans) from 07:00-19:00 h, during which, operative temperatures in open environment were also measured.** The smoothing line is from a generalised additive model.

A temperature-dependent activity rate curve based on the scan surveys (*N*=1127) and T_e_ in the open microhabitat showed that lizards were active and outside their burrows between the operative temperatures of 31.2°C and 48°C. Activity was highest at 39.6°C which is only 0.2°C less than the T_opt_ and within the T_set_ range (see Fig. S5). Foraging in these field sites (*N*=258 observations) occurred between 34.1°C to 48.6°C with the highest proportion of individuals observed foraging at 41.7°C (Fig. 2). The range of T_b_ at which the proportion of individuals foraging is greater than or equal to 95% of maximum proportion of individuals foraging (F_95_) was 40.7°C to 43°C.

### Preferred temperature (T_set_)

Preferred temperature (T_set_) of *S. hardwickii*, from measurements in a thermal gradient in the lab was 39.2°C±0.3°C s.e. and the T_set_ range was 38.2°C±0.3 s.e. –40°C±0.4°C s.e. There was no significant effect of sex (*F(1,14)*= 0.04, *P=*0.84) or BCI (*F(1.14)=*1.19, *P=*0.29 on T_set_.

### Critical thermal thresholds (CT_max_ and CT_min_)

The CT_max_ and CT_min_ of the species was 49°C±0.2°C s.e. and 12.7°C±0.27°C s.e. respectively. There was no significant effect of sex (*F(1,12)=*0.07, *P=*0.79) or BCI (*F(1,12)=*0, *P=*0.97) on CT_max_ of lizards. There was no effect of sex (*F(1,12)=*1.64, *P=*0.22) or BCI (*F(1,12)=*0.89, *P=*0.36) on CT_min_.

### Locomotor performance

**Fig. 3.**
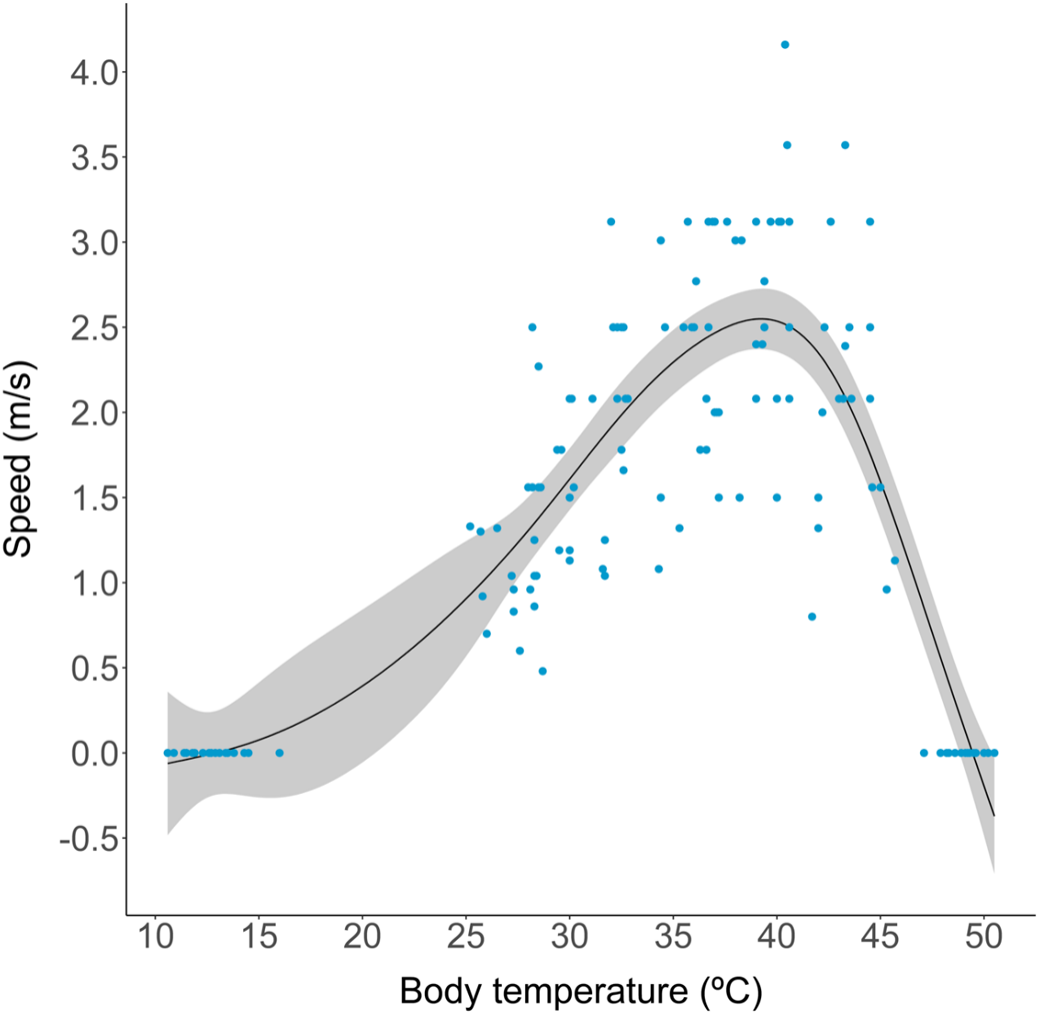
**Temperature-dependence of sprint speed in *Saara hardwickii*, anchored at CT_min_ and CT_max_ where sprint speed is 0 m/s.** The smoothing line is from a generalised additive mixed model (*n*=23 lizards) that also included BCI, sex, site (fixed effects) and individual ID (random effect).

As expected, the thermal performance curve showed a significant effect of temperature on sprint speed of *S. hardwickii* (GAMM: *F(6.27,132.81)*=73.73, *P*<0.001; Fig. 3). The GAMM with temperature as a fixed effect, ‘individual’ as random effect, BCI, sex and site as covariates explained 81.5% of the deviance. The effect of BCI (*β=*0.004, *F(1,132.81)*=6.5, *P*=0.01), sex (*β=*0.3, *F(1,132.81)=*11.2, *P*=0.001) and site (*β=*−0.4, *F(1132.81)=*8.5, *P*=0.004) on sprint speed were weak but statistically significant. The random effect of ‘individual’ (GAMM: *F(0.92,132.81)*=11.72, *P*<0.001) was also significant. Maximum speed (P_max_) was determined to be 2.54 m/s±0.09 s.e. at 39.2°C (T_opt_). The T_opt_ for sprint speed fell within the T_set_ range 38.15°C±0.3°C s.e. −40°C±0.4°C s.e. B_95_ for sprint speed ranged from 36.5°C to 41.5°C.

### Comparing thermal sensitivity of *S. hardwickii* to thermal variability of the environment

**Fig. 4.**
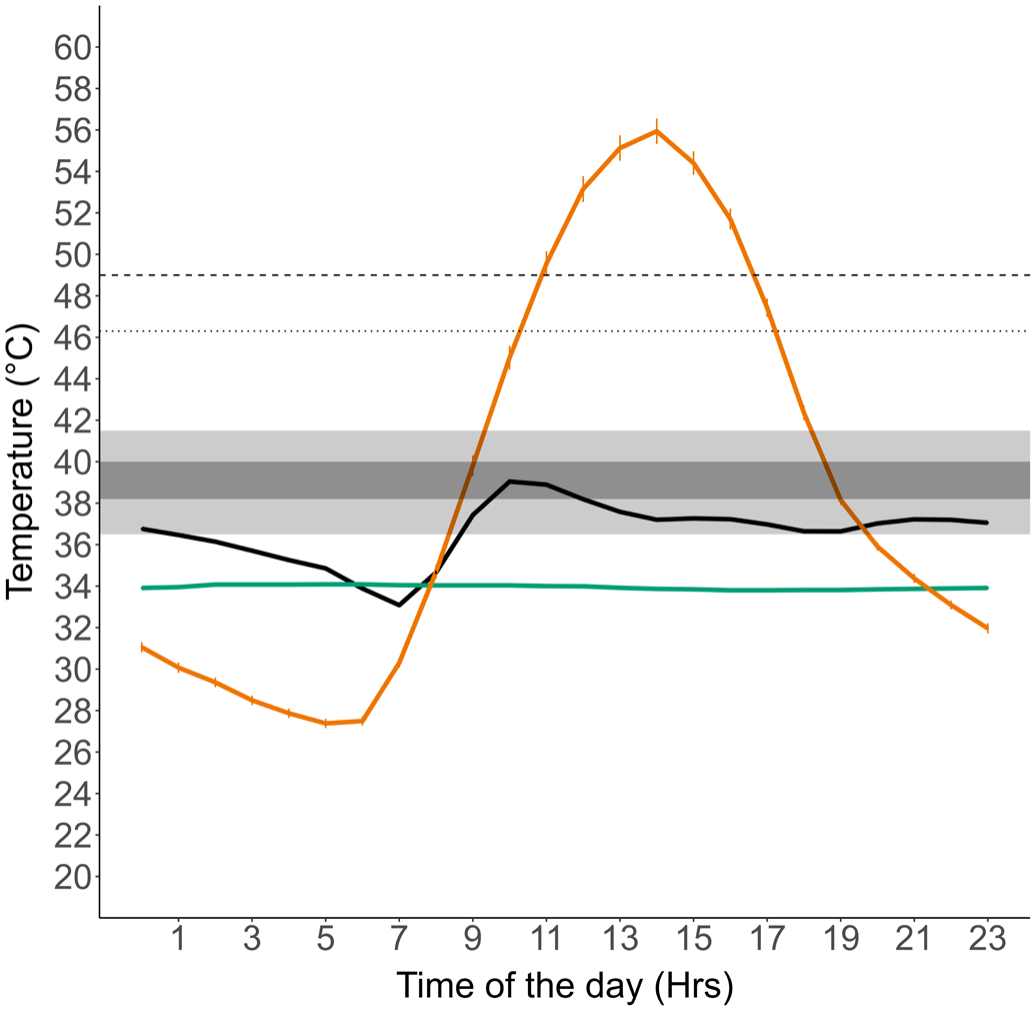
**Average daily temperatures of field body temperatures (T_b_) of *Saara hardwickii* (black line) and operative temperatures (Te) from biophysical copper models in the open environment (orange line) and inside burrows (green line) during the hottest month (May).** Error lines indicate SE. Shown also are thermal thresholds: critical thermal maxima from the lab (CT_max_; dashed line), voluntary thermal maxima from the field (VT_max_; dotted line), B_95_ range (light grey box), and preferred temperature range (T_set_; dark grey box).

Burrow temperatures did not exceed 36.6°C, which is considerably lower than T_set_, B_95_, VT_max_ and CT_max_ (Fig. 4). Temperatures in open environment (Open T_e_) exceeded all thermal limits (T_set_, B_95,_ VT_max_ and CT_max_) for 6 h of the active period of the day (07:00–20:00) in the hottest month of the year (May), during which *S. hardwickii* is restricted to their burrows (Fig. 4).

### Projecting the impact of climate warming

**Fig. 5.**
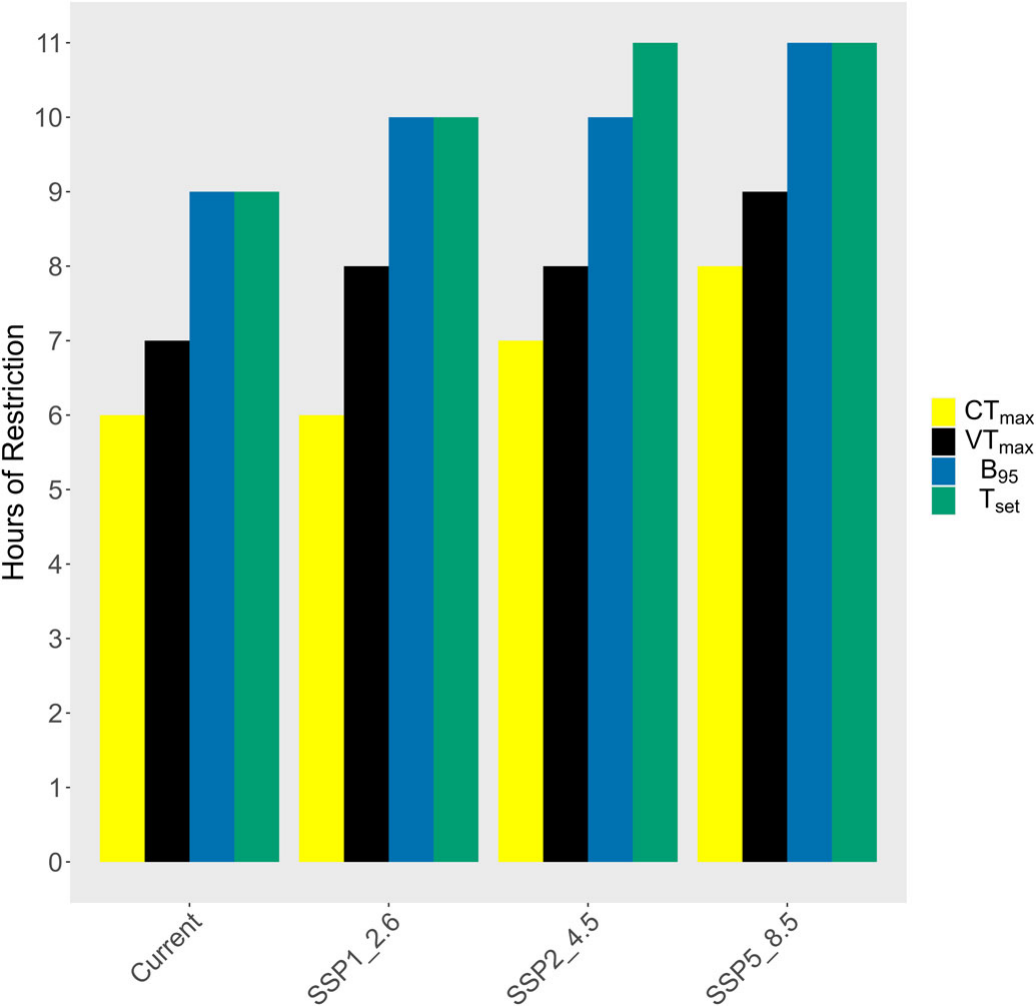
**The number of active-period hours (07:00–20:00) that *Saara hardwickii* is restricted from being in the open, calculated as hours in which environmental temperatures exceed CT_max_ (yellow bars), VT_max_ (black bars), B_95_ (blue bars) and T_set_ (green bars).** Shown are hours of restriction under current conditions, and in the future, based on SSP1_2.6, SSP2_4.5, and SSP5_8.5 climate warming scenarios. Not shown are projections for the burrow as they are thermally buffered with no hours of restriction for any measure of the thermal limits and under any climate warming scenario.

Projection models for h_r_ considering the CT_max_ limit (49°C), showed that there might be an additional 1-h restriction in activity due to climate warming in the intermediate-case (SSP2-4.5) scenario and a 2-h activity restriction in the worst-case (SSP5-8.5) scenario (Fig. 5). Projection models for h_r_, considering the VT_max_ limit (46.3°C), showed that there might be an additional 2-h activity restriction in the best-case (SSP1-2.6) and intermediate-case (SSP2-4.5) scenarios and an additional 3-h restriction in the worst-case (SSP5-8.5) scenario (Fig. 5). Considering the upper bound of B_95_ range of sprint speed (41.5°C), projection models for h_r_ showed that there might be an additional 1-h activity restriction in the best-case (SSP1-2.6) and intermediate-case (SSP2-4.5) scenarios and an additional 2-h restriction in the worst-case (SSP5-8.5) scenario (Fig. 5). Given the upper bound of T_set_ range (40°C), h_r_ projections suggest an additional 1-h activity restriction in the best-case (SSP1-2.6) scenario, and an additional 2-h restriction in intermediate-case (SSP2-4.5) and worst-case (SSP5-8.5) scenarios (Fig. 5). We did not use CT_min_ for our predictions since the difference between the average daily minimum T_b_ and CT_min_ were large (14.8°C).

**Fig. 6.**
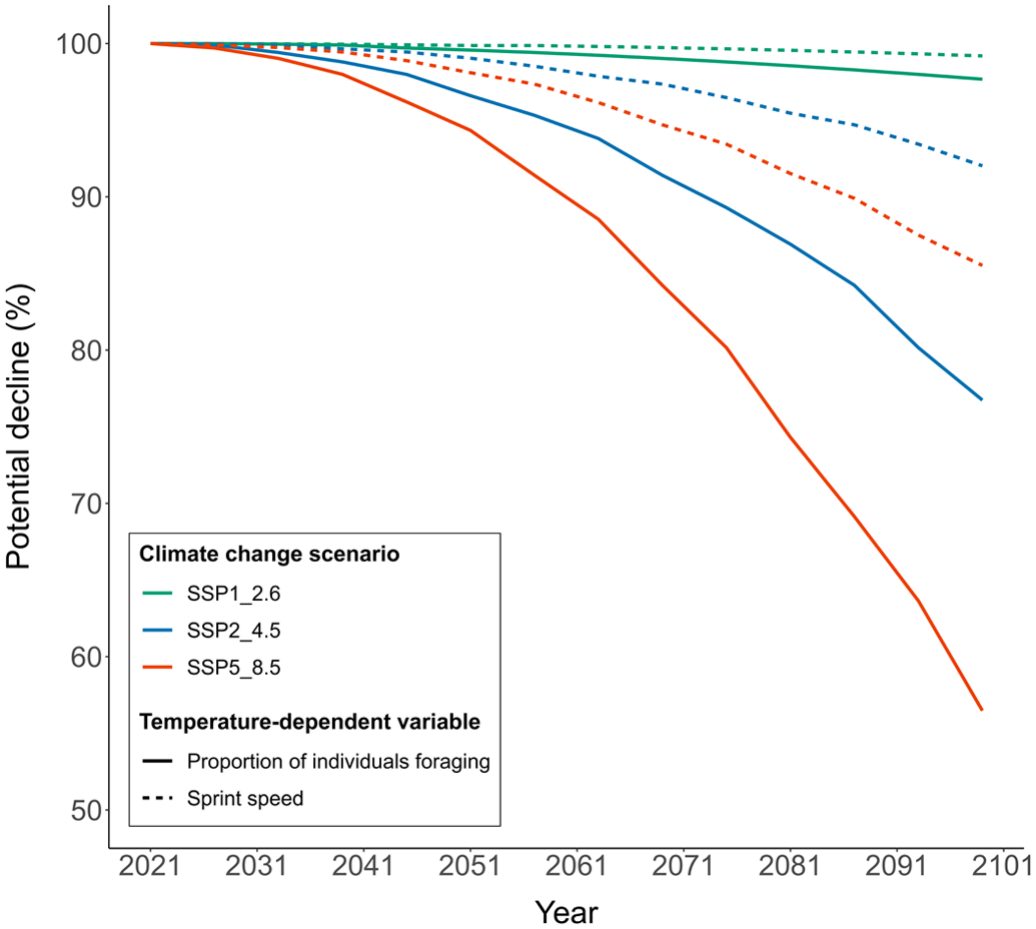
**Potential decline (in %) in sprint speed (dotted lines) and proportion of individuals foraging (solid lines) under different climate change scenarios.** Shown are the future declines based on SSP1_ 2.6 (green lines), SSP2_4.5 (blue lines), and SSP5_8.5 (orange lines) climate change scenarios. Note that the rate of decline is higher for foraging activity in the wild than sprint speed under each of these scenarios.

Projection models suggested a decrease in locomotor performance by 0.8%, 8% and 14.5% in the best-case (SSP1-2.6), intermediate (SSP1-4.5), and worst-case (SSP1-8.5) scenarios respectively by 2099 (Fig. 6). For foraging activity, our model suggests a decrease of 2.3%, 23.2% and 43.5% in the best-case (SSP1-2.6), intermediate (SSP1-4.5), and worst-case (SSP1-8.5) scenarios by 2099 (Fig. 6).

## DISCUSSION

Many animals have physiological, behavioural, or life history strategies that could mitigate the impacts of climate change on fitness (Hoffmann and Sgrò, 2011), yet changes in global temperature are still expected to be a challenge (Parmesan, 2006). Impacts of climate change are thought to be even more pronounced for ectotherms surviving in thermally extreme areas, such as some deserts, where species are already thermally constrained and where thermal buffers for behavioural thermoregulation, such as vegetation, are limited (Grant and Dunham, 1988). In our study on the thermal ecology of *S. hardwickii***,** we integrated lab-based information on thermal responses and limits with field measures of thermal ecology and behaviour to understand how this desert ectotherm thermoregulates and the ways in which it may respond to rising temperatures. Our results support the premise that different physiological and behavioural functions have different thermal optima. Congruent with our broad expectation, we find that projected temperature increases will significantly restrict lizards to the thermal refuges of burrows and substantially decrease both locomotor performance and foraging activity by the end of the century.

Many desert ectotherms, including other spiny-tailed lizard species in the *Uromastyx* genus, rely on burrows to provide thermal relief and refuge from risks (Heatwole, 1970; Mohammed and Hammad, 2008; Wilms et al., 2011; Moore et al., 2018). We find that *S. hardwickii* is a moderately effective thermoregulator and uses burrows and open microhabitats to thermoregulate to maintain their average hourly T_b_ within their T_set_ and/or B_95_ range. We also saw shifts in effectiveness of thermoregulation (E) as the monthly temperatures increased, suggesting an increase in energy used for thermoregulation as the months progressed. As a result, we find that the lizards in these study areas are already restricted to their burrows for 6-9 h out of 13 daylight hours during the hottest month (May) of the year. Burrows provide an excellent buffer for lizards from temperatures in the open (Heatwole, 1970; Ivey et al., 2020; Moore et al., 2018), and for *S. hardwickii* in the Thar desert where vegetation is scant and provide little to no shade, burrows are an essential thermal buffer against extreme temperatures throughout the day. We found that *S. hardwickii* usually emerged out of their burrows after 08:00 h and were mostly in the open until their body temperature (T_b_) reached preferred temperature (T_set_). After approximately 11:00 h, when T_e_ open exceeded VT_max_, body temperature (T_b_) of lizards decreased, which signalled either complete restriction to burrows or higher rates of shuttling between the open microhabitat and burrows (Cowles and Bogert, 1944; personal observations by the authors). *Saara hardwickii* might also be thermoregulating by adjusting body posture (Muth, 1977) or through changes in skin colouration (Dutta and Jhala, 2007), which affects reflectivity and radiant heat gain (Stuart-Fox et al., 2017). Although *S. hardwickii* shows active thermoregulation to avoid extreme body temperatures, the narrow range of body temperatures that we have measured in the wild will have considerable influence on physiological and behavioural performance.

Effective locomotory performance and foraging behaviour are critical to the survival of species, and both are influenced by temperature. Thermal performance measures of *S. hardwickii* indicate their highest sprint speeds at 39.2°C, which is also close to the preferred active temperature for this species in the wild. Fastest sprint speed during the highest activity phase that we document here would be beneficial for effective escape from both terrestrial and avian predators, especially since lizards seldom range far from their burrows. High performance capacity, however, might not imply high activity rates for other behaviours (Gunderson and Leal, 2016). While the total activity of *S. hardwickii* in the wild was highest near its optimal performance temperature (T_opt_), as measured by sprint speed in the lab, and was within its thermal preference range (T_set_), we saw the highest number of individuals foraging in the wild when temperatures were 2.5°C higher than T_opt_. Also, lizards were actively foraging within a narrower temperature range and had higher body temperatures while foraging than when at peak locomotor performance. One possible explanation for these differences in thermal sensitivity of foraging and locomotion is diet. Adults of *S. hardwickii* maintain a primarily herbivorous diet (Dutta and Jhala, 2007) and digestion of plant material is more efficient at higher temperatures (see Qu et al., 2011; Huey, 1982). *Uromastyx aegyptei*, a lizard closely related to *S. hardwickii*, does not chew its food and depends entirely on microbial fermentation for digestion (Foley et al., 1992). Similarly, during our scan surveys, we observed that *S. hardwickii* also does not chew, although information about digestive processes remains unknown. For herbivorous lizards, such as *S. hardwickii*, the possible benefits of high temperature may be negated if increases in environmental temperature forces body temperatures to be even closer to their upper thermal thresholds, resulting in a risk of overheating and a reduction in available activity hours.

We projected the impacts of rising temperatures on *S. hardwickii* using a three-pronged approach. Firstly, we used the ‘activity restriction model’ or ‘threshold model’ posited by Sinervo et al. (2010) to predict the number of hours for which this species would be restricted to their burrows under different climate change scenarios. Our activity restriction models predict that, as temperatures rise, *S. hardwickii* will continue to lose hours of activity during its active months because operative temperatures (T_e_) in the open environment will exceed preferred temperature (T_set_) for the species. Further, T_e_ in the open environment will also exceed B_95_ (the range of T_b_ at which locomotory performance is greater than or equal to 95% of lizard's maximum speed), and both voluntary thermal maxima (VT_max_) in the field and critical thermal maxima (CT_max_). Notably, even in the worst-case climate warming scenario, spiny-tailed lizard burrows in the Thar desert do not seem to exceed thermal thresholds and may be the only microhabitat available to the species as a thermal refuge. In fact, our measure of the thermal refugia in burrows may be an underestimate as we placed copper models only up to 1 m inside burrows, but temperatures could be even cooler deeper within burrows. We included T_set_ and B_95_ in our model because outside the critical threshold ranges (CT_max_), preference or performance are compromised and may also affect survival probability. This conservative measure allows us to plan conservation efforts before critical thermal limits are reached. During our observations of lizard activity, we recorded lizards using burrow entrances for territory defence and foraging. However, the projected levels of warming might restrict lizards to deeper parts of the burrow for more hours, further reducing their time to forage, defend territories, and obtain mating opportunities which occur in the open environment. Further studies exploring the thermal gradient of the species’ burrows are required, but since burrows are not used for active foraging or mating, the climate-induced increases in burrow use will still come at a cost for the species in this landscape.

Rising temperatures will not only influence the number of hours that are thermally suitable for ectothermic species but will affect body performance and temperature-sensitive activity. Consistent with our expectation, we observed a notable reduction in locomotor performance of *S. hardwickii* under global warming conditions. Reduction in sprint speed that range from 0.8% to 14.5% by 2099, depending on the climate projection model, might have a significant negative impact on the species’ ability to evade predators during their active hours (Ekner-Grzyb et al., 2013; Husak, 2006). Physiological thresholds and performance do not necessarily govern the activity of all individuals, and thus we expect some variation in the number of individuals that are active, depending on environmental temperatures as well as on individual variation (Gunderson and Leal, 2016). Since this study considers the impacts of climate change through the lens of the Bogert effect, our projections, like many others, do not take into account genetic adaption or plasticity of thermal thresholds, which may play an important role in increasing the odds of survival for *S. hardwickii* (Gunderson and Leal, 2012). These projections also do not incorporate potential changes in the forage availability or vegetation structure in the environment. When we modelled changes in the proportion of individuals foraging solely as a function of environmental temperature, we found that the reduction in foraging activity was even more dramatic than the reductions in locomotor performance. In the worse-case global warming scenario, the number of individuals foraging is projected to reduce by 43.5%, which might prove to be devastating for an herbivorous lizard in terms of survival. If these temperature-induced reductions in foraging activity are coupled with temperature-induced reduction in forage availability, *S. hardwickii* becomes even more vulnerable.

Many studies have used physiological parameters, such as thermal thresholds and locomotor performance, to predict the impacts of climate change on ectotherms (e.g. Gunderson and Leal, 2012; Huey et al., 2009; Ivey et al., 2020; Logan et al., 2013; Wilms et al., 2011). It is also well known that ectotherms can behaviourally thermoregulate to optimize their performance within an optimum temperature range in the wild, despite heterogeneity and change in environmental conditions. Active behavioural regulation can, in fact, constrain rather than drive evolution of thermal traits (i.e. the Bogert effect; Huey et al., 2003). However, when the temperature range for activity is narrower than the physiological limits, as we see for *S. hardwickii*, using physiological constraints and not accounting for activity in the wild might underestimate the impacts of climate change (Gunderson and Leal, 2016). On the flipside, using activity in the wild could also overestimate the impacts of climate change as behaviour is more plastic than thermal thresholds. However, we found the thermal optima of locomotory performance and the operative temperature at which total activity in the wild is highest is very similar. This suggests that even though the thresholds for activity in the wild might be more plastic, a decline in activity from optimum when environmental temperature increases is still expected. Overall, key data on thermal ecology with information about temperature-sensitive behaviours in the wild is necessary to understand the ways in which increasing temperatures may influence ectothermic vertebrates, especially for vulnerable species such as S. *hardwickii*, which need climate-aware conservation plans and policies for their continued protection.

## MATERIALS AND METHODS

### Study area

All sampling and data collection were conducted at two sites in the Thar desert of Jaisalmer: Sam (26°49′32.5″N 70° 30′42.3″E) and Bedhiya (26°52′07.3″N 70°27′36.6″E) near Desert National Park Wildlife Sanctuary, India (see Fig. S1 for images of field sites). These areas are characterized by extremely harsh, arid summers with air temperatures reaching up to 50°C, and cold winters with temperatures dropping as low as 0°C. Rainfall in the Thar landscape is erratic and typically ranges between 100 and 450 mm per year (weather station data, Jaisalmer; ∼45 km from both sites). Data on the thermal ecology of this species were generated between a relatively cooler month (March) and the hottest month (May) within their active season in 2021.

### Operative temperatures in the wild (T_e_)

To quantify the operative environmental temperature (T_e_) of lizards in different microhabitats, we deployed biophysical models made from copper in areas that lizards use (as per Bakken, 1992; Bakken and Angilletta, 2014; Dzialowski, 2005). Copper models were moulded to match a typical adult, including the tail which is large and does not autotomize in this species (400 mm length×35 mm width). Models were hollow but closed on both sides, with a temperature logger (Maxim Thermochron iButton DS1923; accuracy±0.5°C) positioned in the middle using a plastic ring. The loggers were set to record operative temperature every 15 min for 3 months. To determine the appropriate size of copper model for T_e_ measurements, we tested two sizes that emulated adult and subadult lizards (See Fig. S2 for details). We found no significant differences in T_e_ between these copper models and thus only the adult size was used for the rest of the analyses. Copper model temperatures were also calibrated in the laboratory by comparing them with cloacal and skin temperatures of three wild-caught lizards of different size classes (see Fig. S3).

*Saara harwickii* in the Thar landscape mainly use the open environment and burrows in these study areas. Although trees and shrubs are present at the Bedhiya study site, the lizards do not typically use them for thermoregulation. Thus, to capture the variation in T_e_ between the most extreme microhabitats, we positioned copper models in the open and within burrows. For T_e_ in the open microhabitats, copper models (moulded with body+legs) were positioned in direct sunlight (*n*=2). For T_e_ within burrows, copper models (body only) was placed ∼1 m inside an inactive burrow (*n*=2). Copper models that resembled only the body of a lizard was used within the burrow because burrows are narrow and lizards come into direct contact with the burrow walls. Furthermore, we expect heat transfer within burrows to be via conduction whereas on the surface, heat transfer happens via conduction, solar radiation, radiation from the ground, and convection (Muth, 1977). We used only four copper models per site (Sam and Bedhiya) as the study sites were relatively homogeneous in terms of microhabitat structure. Within each site, copper models were placed 500 m apart.

To evaluate the factors affecting T_e_, we used a linear model (LM) with T_e_ as the response variable, and microhabitat (open or burrow), site (Sam and Bedhiya) and month (March, April, and May) as predictor variables with microhabitat and month as interaction terms. We used scatter plots to evaluate linearity and Q-Q plots to check for the normality of residuals.

### Field active body temperature (T_b_) and effectiveness of thermoregulation

To measure the body temperatures (T_b_) of lizards in the field, 25 individuals were captured with a lasso at their burrow entrance and were individually marked using a nontoxic felt-tip permanent marker (Johnson, 2005). Mass (Pesola^®^ 500 g precision scale, ±0.5 g), snout–vent length (SVL, ±0.1 cm), sex, and reproductive status if female (using abdominal palpations) were recorded for all individuals. If the captured lizard was a gravid female, we released it at the burrow from which we had initially captured it. Mass and SVL were used to calculate body condition index (BCI; as per Cecchetto et al., 2020; Peig and Green, 2009).

Each lizard was then fitted with a breathable cotton harness that had a temperature data-logger (Maxim Thermochron iButton DS1921G; accuracy ±0.5°C) stitched into it, such that the sensing surface of the logger touched the skin of the lizard. The combined weight of the logger and the harness was <6 g (<5% of lizard body weight), and the harness allowed free movement of the lizard (see Fig. S2 for the image of the harness attached to the lizard). The temperature logger was set to measure skin temperature (T_b_) at 15-min intervals with data ranging from 17 to 63 days between March and May 2021. Field body temperatures (T_b_) from 19 individuals were successfully obtained and data were checked manually for aberrant readings, which were removed (e.g. consecutive readings with a 10°C difference suggested detachment of the thermal logger). Lizard skin temperature (T_b_) was used as a proxy for core temperature, which we validated in the lab (see Fig. S3). For all lizards (*n*=19), voluntary thermal maximum T_b_ (VT_max_) was calculated as the average maximum daily T_b_ (Ivey et al., 2020; Brattstrom, 1965).

To evaluate the factors affecting T_b_, we used a linear mixed model (LMM) with field T_b_ as the response variable, and open T_e_, BCI, and sex as fixed effects. Individual identity was included as a random variable. Scatter plots and Q–Q plots were used to evaluate linearity and normality of residuals respectively.

To quantify the effectiveness of thermoregulation (*E*), we calculated the thermoregulatory accuracy (*d_b_*) and the thermal quality of the habitat (*d_e_*). We measured *db* as the mean of deviations of T_b_ from T_set_ (see below for T_set_ methods). If T_b_ was below the T_set_ range, we subtracted T_b_ from the lower bound of T_set_ range. If T_b_ was above the T_set_ range, we subtracted upper bound of T_set_ from T_b_. We measured *de* as the mean of deviations of T_e_ from all microhabitats from T_set_. If T_e_ was below the T_set_ range, we subtracted T_e_ from the lower bound of T_set_ range. If T_e_ was above the T_set_ range, we subtracted upper bound of T_set_ from T_e_. Using these two measures, we calculated 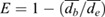, where the overbars depict the mean values of the variables (Hertz et al., 1993; Blouin-Demers and Weatherhead, 2001).

### Temperature-dependent activity

To evaluate the direct effects of temperature on the activity of the lizard, we used a combination of focal observations, scan surveys and T_e_. Behavioural observations were conducted from a hide using binoculars from 07:00 to 19:00 for 10 days (5 days per site) spread over each month (March–May). Overcast and rainy days were avoided.

We used temperature-based activity estimation algorithm (TBAE; Davis et al., 2008; Moore et al., 2018) that evaluated if marked and tagged lizards (*n*=19) were active or inactive, based on the absolute difference between body (T_b_) and operative (T_e_) temperatures. If T_b_ was closer to T_e_ of open environment than that of burrow, then the state was classified as ‘active’, and vice-versa (Moore et al., 2018). Scan sampling was carried out to validate these results, wherein we recorded the activity of marked and tagged individuals (*n*=19) as ‘active’ (in the open) or ‘inactive’ (inside burrow) until the lizard was out-of-sight (Altmann, 1974). We compared the activity budget created by the above algorithm with that obtained from focal observations, and the accuracy of the estimated activity budgets was assessed using Cohen's Kappa test (Davis et al., 2008).

To assess the temperature dependence of foraging activity in the wild, we conducted scan sampling of lizards from 10 active *S. hardwickii* burrows that were randomly chosen and marked with flags. Scan sampling of lizards emerging from these burrows was carried out at 15-min intervals from 07:00 h to 19:00 h, and proportion of individuals foraging at any scan were computed. We used generalized additive model (GAM) to predict foraging activity as a function of Open T_e_ using the package ‘mgcv’ (Wood and Wood, 2015) in R version 4.1.0 (R core team, 2020) as the expected relationship between temperature and activity was non-linear and unimodal. We also calculated the range of T_b_ at which the proportion of individuals foraging is greater than or equal to 95% of maximum proportion of individuals foraging (F_95_).

### Housing conditions in the lab

Measures of preferred body temperature (T_set_), critical thermal maxima (CT_max_) and minima (CT_min_), and locomotor performance were conducted at the field station. For these measurements, adult lizards within the size range of 14.5 to 24.2 cm SVL were captured from the wild and housed in individual terraria (62×62×30 cm) that had a 150W heating lamp and artificial refugia made of untreated wood. During captivity, lizards were provided with *ad libitum* access to food (fresh and washed *Dactyloctenium aegyptium* leaves). All experiments described below were carried out in a cool room (22.5–26°C) between 08:00 and 19:00 after acclimation for 24 h. The order of measurements was the following. On the first day, we conducted the T_set_ experiment and obtained two measures of sprint speed at different temperatures separated by a minimum of 4 h of recovery (Angilletta et al., 2002). On the second day, two additional measures of sprint speed at different temperatures were taken and CT_min_ was measured. On the last day, one measure of sprint speed was performed, and CT_max_ was measured at the end. Due to permit restrictions for this protected species, the sample sizes for each measurement were limited to the minimum. To reduce the effects of fatigue, not all captured lizards (*N*=23) were used for both T_set_ and critical thermal measurements. Capture was staggered such that lizards spent no more than 4 days in captivity. To ensure that time in captivity and the experiments did not adversely affect the performance of lizards, we measured the mass of all lizard before and after the experiments and found no significant changes [*T*(44)=−0.03, *P*=0.97]. Thereafter, lizards were released near their respective burrows.

### Preferred Body Temperature (T_set_)

To measure T_set_, we created a thermal gradient in the lab which comprised of a long and narrow wooden frame (300×25×25 cm) with a sandpaper substrate, and a 150W infrared lamp at one end, which generated a temperature gradient from 25°C to 52°C. Each lizard (*N*=7 female, 10 male) was introduced individually to the gradient and allowed to acclimatize for 2 h, after which, body (cloacal) temperatures were recorded every 5 min for an hour. Cloacal temperature was measured using a temperature logger (Amprobe model TMD-50) that was connected to a k-type thermocouple which was inserted 1 cm into the cloaca of the lizard and kept in place using surgical tape. Temperatures were logged automatically. T_set_ was considered as the 25–75% interquartile range of the cloacal temperatures of the last hour of the 3-h trial. We excluded T_set_ data for the lizards which had their T_set_ >2 SD away from the mean of the median T_set_ of all the lizards, or those who had failed to move and actively thermoregulate within the gradient (Ivey et al., 2020). A linear model (LM) was used to examine the effects of BCI and sex on T_set_. We ensured the normality of residuals using the Shapiro–Wilk test (*w*=0.44, *P*=0.94) and linearity using residuals versus fitted plots.

### Critical thermal maxima (CT_max_) and minima (CT_min_)

We measured CT_max_ and CT_min_ on 15 individuals (*N*=7 female, 8 male). CT_max_ was the body temperature at which onset of muscle spasms was observed and CT_min_ was the body temperature at which loss of righting response (ability to turn over when placed on its back) was observed_._ During the trials, cloacal temperature was measured using a temperature logger (Amprobe model TMD-50) that was connected to a k-type thermocouple which was inserted 1 cm into the cloaca of the lizard and kept in place using surgical tape. The initial body temperature of the lizards before the experiments was ∼25°C. To determine CT_max_, each lizard was placed in a metal container with a 150W infrared lamp 40 cm above it, which resulted in a heating rate of approximately 1°C per min for the lizard. Cloacal temperatures and behaviour were constantly monitored, and CT_max_ was recorded as the cloacal temperature at which muscle spasms were first observed (Lutterschmidt and Hutchison, 1997a,b). Once lizards reached CT_max,_ they were immediately removed from the container and transferred to a pre-cooled chamber to assist in lowering their body temperature. To determine CT_min_, each lizard was placed in a terrarium in a room at 25°C. Using icepacks, the body temperature of the lizard was lowered at a rate of approximately 0.5°C min^−1^. We started testing the righting response of the lizard when the terrarium temperature reached 8°C. Righting response was tested every 15 s until the lizard could not right itself when flipped on its back (Labra and Bozinovic, 2002; Laspiur et al., 2021). The terrarium temperatures were reduced to as low as 2°C. When CT_min_ was reached, the lizard was placed at room temperature. No lizards died during these experiments, and all resumed normal activity (movement and feeding) within 2–5 h after these experiments. We used linear models (LM) to statistically determine the effects of BCI and sex on CT_max_ and CT_min_. We ensured the normality of residuals using the Shapiro–Wilk test (CT_min_; *w*=0.91, *P*=0.14, CT_max_; *w*=0.95, *P*=0.66) and linearity using residuals versus fitted plots.

### Locomotor performance

Locomotor performance for each lizard (*N*=12 female, 11 male) was measured at five body temperatures between 25°C and 45°C, with approximately 5°C intervals. Body temperatures above 34°C were achieved using the method described for CT_max_, and a water bath with icepacks was used to reduce body temperature of lizards. Each lizard was kept at the selected temperature for 1 h before introducing it to a racetrack (250×25×25 cm). The racetrack was made of a wooden frame lined with 260-grit sandpaper to emulate the most common substrate in the wild: fine gravel (Ramesh and Ishwar, 2008). Each lizard was made to run two sprint trials per day separated by >4 h. The trial run consisted of five consecutive 0.5 m sections, with a single initial stimulus (touching their rear thigh with a finger). This was repeated three times, separated by five-minute rest. Nikon D7000 camera with Tokina 11–16 mm lens was used to record all trials at 25 frames per second. All videos were analysed in Avidemux 2.7.8 and the fastest run for each lizard was used for the analysis. A trial was not included in the analysis if the lizard failed to move.

Optimum temperature (T_opt_) was considered to be the T_b_ (treatment body temperatures of the lizards) at which the lizard's speed was maximum (P_max_). Performance breath (B_95_) was defined as the range of T_b_ at which the performance is greater than or equal to 95% of P_max_ (Cecchetto et al., 2020). To calculate these variables, generalized additive mixed models (GAMMs) were fitted to the non-linear data using the package ‘mgcv’ (Wood and Wood, 2015) in R 4.1.0 (R core team, 2020) where T_b_, BCI, sex and site were considered as fixed effects, after accounting for the random variation between individuals which was expected to be significant (Artacho et al., 2013; Cecchetto et al., 2020). We included CT_max_ and CT_min_ data to anchor values for body temperatures, at which sprint speed is 0 m/s (similar to Cecchetto et al., 2020). A coARMA correlation structure with two knots were used to construct the curve.

### Projecting the impact of climate warming

We projected the impact of climate warming on hours of restriction (h_r_), locomotor performance and foraging activity. We used projections based on three future pathways of societal development: shared socioeconomic pathways (SSPs), (1) SSP1-2.6, where there would be an increase of 1°C by 2099 (+0.011°C per year; 2099 represented by the time slice 2080–2099); (2) SSP2-4.5, where there would be an increase of 3°C by 2099 (+0.036°C per year) and (3) SSP5- 8.5, where there would be an increase of 4°C by 2099 (+0.047°C per year). These SSPs align with the projections for Jaisalmer based on the ensemble of 27 coupled model intercomparison project phase 6 (CMIP6) climate models available for temperature (https://esgf-node.llnl.gov/search/cmip6). Before the ensemble, models were re-gridded to 1°×1° resolution from their original resolution using a bilinear interpolation method (Almazroui et al., 2020). The projected increments in temperature were added to the T_e_ data.

Activity restriction in different microhabitats (open and burrow) was projected using CT_max_, VT_max_, B_95_ and T_set_ as different thresholds for activity. The number of hours above the different thermal thresholds in each microhabitat were considered as the hours of restriction (h_r_), and we projected the h_r_ for each threshold variable by adding the projected increase in temperature to current biophysical model data unilaterally. It is likely that different microhabitats will have different rates of temperature increase but this model will provide a broad estimate of how h_r_ might change with climate warming (Brusch et al., 2016).

To project the future reductions in foraging opportunity, the current maximum proportion of individuals foraging (based on the scan survey data) at the associated T_e_ was set to 100%. By adding the projected temperature increases to the curve of foraging-temperature dependence (constructed using GAM), we calculated the potential decline in foraging opportunity in the future. These predictions are restricted to summer when the temperatures are the highest in the desert ecosystem of Jaisalmer. To project changes in locomotor performance in warming conditions, current performance at optimal temperature (P_max_) was set to 100% and increases in temperature on the thermal performance curve (constructed using GAMM) were used to calculate potential relative decrease in future.

## Supplementary Material

10.1242/biolopen.060150_sup1 Supplementary information

## References

[BIO060150C1] Abram, P. K., Boivin, G., Moiroux, J. and Brodeur, J. (2017). Behavioural effects of temperature on ectothermic animals: Unifying thermal physiology and behavioural plasticity. *Biol. Rev. Camb. Philos. Soc.* 92, 1859-1876. 10.1111/brv.12312 28980433

[BIO060150C2] Almazroui, M., Saeed, S., Saeed, F., Islam, M. N. and Ismail, M. (2020). Projections of precipitation and temperature over the south asian countries in CMIP6. *Earth Syst. Environ.* 4, 297-320. 10.1007/s41748-020-00157-7

[BIO060150C3] Altmann, J. (1974). Observational study of behavior: Sampling methods. *Behaviour* 49, 227-266. 10.1163/156853974X00534 4597405

[BIO060150C4] Angilletta, M. J. (2009). *Thermal Adaptation: A Theoretical and Empirical Synthesis*. Oxford, UK: Oxford University Press.

[BIO060150C5] Angilletta, M. J., Niewiarowski, P. H. and Navas, C. A. (2002). The evolution of thermal physiology in ectotherms. *J. Therm. Biol.* 27, 249-268. 10.1016/S0306-4565(01)00094-8

[BIO060150C6] Artacho, P., Jouanneau, I. and Le Galliard, J.-F. (2013). Interindividual variation in thermal sensitivity of maximal sprint speed, thermal behavior, and resting metabolic rate in a lizard. *Physiol. Biochem. Zool.* 86, 458-469. 10.1086/671376 23799840

[BIO060150C7] Bakken, G. S. (1992). Measurement and application of operative and standard operative temperatures in ecology. *Am. Zool.* 32, 194-216. 10.1093/icb/32.2.194

[BIO060150C8] Bakken, G. S. and Angilletta, M. J. (2014). How to avoid errors when quantifying thermal environments. *Funct. Ecol.* 28, 96-107. 10.1111/1365-2435.12149

[BIO060150C9] Blouin-Demers, G. and Weatherhead, P. J. (2001). Thermal ecology of Black Rat Snakes (*Elaphe obsoleta*) in a thermally challenging environment. *Ecology* 82, 3025-3043. 10.1890/0012-9658(2001)082[3025:TEOBRS]2.0.CO;2

[BIO060150C10] Booth, T. H., Nix, H. A., Busby, J. R. and Hutchinson, M. F. (2014). BIOCLIM: the first species distribution modelling package, its early applications and relevance to most current MAXENT studies. *Divers. Distrib.* 20, 1-9. 10.1111/ddi.12144

[BIO060150C11] Brattstrom, B. H. (1965). Body temperatures of reptiles. *Am Midl Nat* 73, 376-422. 10.2307/2423461

[BIO060150C12] Briscoe, N. J., Kearney, M., Taylor, C. A. and Wintle, B. A. (2016). Unpacking the mechanisms captured by a correlative species distribution model to improve predictions of climate refugia. *Glob. Change Biol.* 22, 2425-2439. 10.1111/gcb.13280 26960136

[BIO060150C13] Brusch, G. A., Taylor, E. N. and Whitfield, S. M. (2016). Turn up the heat: Thermal tolerances of lizards at La Selva, Costa Rica. *Oecologia* 180, 325-334. 10.1007/s00442-015-3467-3 26466592

[BIO060150C14] Burraco, P., Orizaola, G., Monaghan, P. and Metcalfe, N. B. (2020). Climate change and ageing in ectotherms. *Glob. Change Biol.* 26, 5371-5381. 10.1111/gcb.15305 32835446

[BIO060150C15] Camacho, A. and Rusch, T. W. (2017). Methods and pitfalls of measuring thermal preference and tolerance in lizards. *J. Therm. Biol.* 68, 63-72. 10.1016/j.jtherbio.2017.03.010 28689723

[BIO060150C16] Cecchetto, N. R., Medina, S. M. and Ibargüengoytía, N. R. (2020). Running performance with emphasis on low temperatures in a Patagonian lizard, *Liolaemus lineomaculatus*. *Sci. Rep.* 10, 14732. 10.1038/s41598-020-71617-3 PMC7477221 32895421

[BIO060150C17] Cowles, R. B. and Bogert, C. M. (1944). A preliminary study of the thermal requirements of desert reptiles. *Bull. Am. Mus. Nat. Hist.* 83, 261-296.

[BIO060150C18] Das, S. K., Dookia, S., Das, K. and Dutta, S. K. (2013). Ecological observations on the Indian Spiny-tailed Lizard *Saara hardwickii* (Gray, 1827) (Reptilia: Squamata: Agamidae) in Tal Chhapar Wildlife Sanctuary, Rajasthan, India. *Journal of Threatened Taxa* 5, 3516-3526. 10.11609/JoTT.o2806.484

[BIO060150C19] Davis, J., Taylor, E. and DeNardo, D. (2008). An automated temperature-based option for estimating surface activity and refuge use patterns in free-ranging animals. *J. Arid Environ.* 72, 1414-1422. 10.1016/j.jaridenv.2008.02.018

[BIO060150C20] Dutta, S. and Jhala, Y. (2007). Ecological aspects of Indian spiny-tailed lizard *Uromastyx hardwickii* in Kutch. *J. Bombay Nat. Hist. Soc.* 104, 255-265.

[BIO060150C21] Dutta, S. and Jhala, Y. (2014). Planning agriculture based on landuse responses of threatened semiarid grassland species in India. *Biol. Conserv.* 175, 129-139. 10.1016/j.biocon.2014.04.026

[BIO060150C22] Dzialowski, E. (2005). Use of operative and standard operative temperature models in thermal biology. *J. Therm. Biol.* 30, 317-334. 10.1016/j.jtherbio.2005.01.005

[BIO060150C23] Ekner-Grzyb, A., Sajkowska, Z., Dudek, K., Gawałek, M., Skórka, P. and Tryjanowski, P. (2013). Locomotor performance of sand lizards (*Lacerta agilis*): Effects of predatory pressure and parasite load. *Acta Ethologica* 16, 173-179. 10.1007/s10211-013-0148-2 PMC3775096 24052686

[BIO060150C24] Foley, W. J., Bouskila, A., Shkolnik, A. and Choshniak, I. (1992). Microbial digestion in the herbivorous lizard *Uromastyx aegyptius* (Agamidae). *J. Zool.* 226, 387-398. 10.1111/j.1469-7998.1992.tb07486.x

[BIO060150C25] Gates, D. M. (2012). *Biophysical Ecology*. Massachusetts, USA: Courier Corporation.

[BIO060150C26] Grant, B. W. and Dunham, A. E. (1988). Thermally imposed time constraints on the activity of the desert lizard *Sceloporus merriami*. *Ecology* 69, 167-176. 10.2307/1943171

[BIO060150C27] Gunderson, A. R. and Leal, M. (2012). Geographic variation in vulnerability to climate warming in a tropical Caribbean lizard. *Funct. Ecol.* 26, 783-793. 10.1111/j.1365-2435.2012.01987.x

[BIO060150C28] Gunderson, A. R. and Leal, M. (2015). Patterns of thermal constraint on ectotherm activity. *Am. Nat.* 185, 653-664. 10.1086/680849 25905508

[BIO060150C29] Gunderson, A. R. and Leal, M. (2016). A conceptual framework for understanding thermal constraints on ectotherm activity with implications for predicting responses to global change. *Ecol. Lett.* 19, 111-120. 10.1111/ele.12552 26647860

[BIO060150C30] Heatwole, H. (1970). Thermal ecology of the desert dragon *Amphibolurus inermis*. *Ecol. Monogr.* 40, 425-457. 10.2307/1942339

[BIO060150C31] Hertz, P. E., Huey, R. B. and Stevenson, R. (1993). Evaluating temperature regulation by field-active ectotherms: The fallacy of the inappropriate question. *Am. Nat.* 142, 796-818. 10.1086/285573 19425957

[BIO060150C32] Hoffmann, A. A. and Sgrò, C. M. (2011). Climate change and evolutionary adaptation. *Nature* 470, 479-485. 10.1038/nature09670 21350480

[BIO060150C33] Huey, R. B. (1982). Temperature, physiology, and the ecology of reptiles. *Biol. Reptilia* 12, 25-91.

[BIO060150C34] Huey, R. B., Hertz, P. E. and Sinervo, B. (2003). Behavioral drive versus behavioral inertia in evolution: A null model approach. *Am. Nat.* 161, 357-366. 10.1086/346135 12699218

[BIO060150C35] Huey, R. B., Deutsch, C. A., Tewksbury, J. J., Vitt, L. J., Hertz, P. E., Alvarez Pérez, H. J. and Garland, T. Jr. (2009). Why tropical forest lizards are vulnerable to climate warming. *Proc. R. Soc. B* 276, 1939-1948. 10.1098/rspb.2008.1957 PMC2677251 19324762

[BIO060150C36] Huey, R. B., Losos, J. B. and Moritz, C. (2010). Are lizards toast? *Science* 328, 832-833. 10.1126/science.1190374 20466909

[BIO060150C37] Husak, J. F. (2006). Does survival depend on how fast you can run or how fast you do run? *Funct. Ecol.* 20, 1080-1086. 10.1111/j.1365-2435.2006.01195.x

[BIO060150C38] IPBES (2019). Summary for policymakers of the global assessment report on biodiversity and ecosystem services of the Intergovernmental Science–Policy Platform on Biodiversity and Ecosystem Services. *Bonn*, *Germany: IPBES Secretariat*. https://Ipbes.net/Global-Assessment-Report-Biodiversity-Ecosystem-Services.

[BIO060150C39] IPCC (2019). Climate Change and Land: an IPCC special report on climate change, desertification, land degradation, sustainable land management, food security, and greenhouse gas fluxes in terrestrial ecosystems [P.R. Shukla, J. Skea, E. Calvo Buendia, V. Masson-Delmotte, H.-O. Pörtner, D. C. Roberts, P. Zhai, R. Slade, S. Connors, R. van Diemen, M. Ferrat, E. Haughey, S. Luz, S. Neogi, M. Pathak, J. Petzold, J. Portugal Pereira, P. Vyas, E. Huntley, K. Kissick, M. Belkacemi, J. Malley, (eds.)]. In press.

[BIO060150C40] Ivey, K. N., Cornwall, M., Crowell, H., Ghazian, N., Nix, E., Owen, M., Zuliani, M., Lortie, C. J., Westphal, M. and Taylor, E. (2020). Thermal ecology of the federally endangered blunt-nosed leopard lizard (*Gambelia sila*). *Conserv. Physiol.* 8, coaa014. 33649711 10.1093/conphys/coaa014 PMC7047230

[BIO060150C41] Johnson, M. (2005). A new method of temporarily marking lizards. *Herpetol. Rev.* 36, 277-279.

[BIO060150C42] Kearney, M. R. (2013). Activity restriction and the mechanistic basis for extinctions under climate warming. *Ecol. Lett.* 16, 1470-1479. 10.1111/ele.12192 24118740

[BIO060150C43] Khan, M. Z. and Mahmood, N. (2004). Study of population status and natural history of agamid lizards of Karachi. *Pak. J. Biol. Sci.* 7, 1942-1945. 10.3923/pjbs.2004.1942.1945

[BIO060150C44] Kingsolver, J. G., Diamond, S. E. and Buckley, L. B. (2013). Heat stress and the fitness consequences of climate change for terrestrial ectotherms. *Funct. Ecol.* 27, 1415-1423. 10.1111/1365-2435.12145

[BIO060150C45] Labra, A. and Bozinovic, F. (2002). Interplay between pregnancy and physiological thermorégulation in *Liolaemus* lizards. *Écoscience* 9, 421-426. 10.1080/11956860.2002.11682729

[BIO060150C46] Laspiur, A., Santos, J. C., Medina, S. M., Pizarro, J. E., Sanabria, E. A., Sinervo, B. and Ibargüengoytía, N. R. (2021). Vulnerability to climate change of a microendemic lizard species from the central Andes. *Sci. Rep.* 11, 11653. 10.1038/s41598-021-91058-w 34079000 PMC8172825

[BIO060150C47] Logan, M. L., Huynh, R. K., Precious, R. A. and Calsbeek, R. G. (2013). The impact of climate change measured at relevant spatial scales: new hope for tropical lizards. *Glob. Change Biol.* 19, 3093-3102. 10.1111/gcb.12253 23661358

[BIO060150C48] Logan, M. L., van Berkel, J. and Clusella-Trullas, S. (2019). The Bogert Effect and environmental heterogeneity. *Oecologia* 191, 817-827. 10.1007/s00442-019-04541-7 31679039

[BIO060150C49] Lutterschmidt, W. I. and Hutchison, V. H. (1997a). The critical thermal maximum: History and critique. *Can. J. Zool.* 75, 1561-1574. 10.1139/z97-783

[BIO060150C50] Lutterschmidt, W. I. and Hutchison, V. H. (1997b). The critical thermal maximum: Data to support the onset of spasms as the definitive end point. *Can. J. Zool.* 75, 1553-1560. 10.1139/z97-782

[BIO060150C51] Mohammed, E. H. A. and Hammad, D. M. (2008). Notes on a sympatric population of two species of spiny-tailed lizards in Sudan: *Uromastyx dispar Heyden*, 1827, and *U. ocellata* Lichtenstein, 1823 (Sauria: Agamidae). *Zool. Middle East* 44, 51-56. 10.1080/09397140.2008.10638288

[BIO060150C52] Moore, D., Stow, A. and Kearney, M. R. (2018). Under the weather?—The direct effects of climate warming on a threatened desert lizard are mediated by their activity phase and burrow system. *J. Anim. Ecol.* 87, 660-671. 10.1111/1365-2656.12812 29446081

[BIO060150C53] Muth, A. (1977). Thermoregulatory postures and orientation to the sun: A mechanistic evaluation for the Zebra-Tailed Lizard, *Callisaurus draconoides*. *Copeia* 1977, 710-720. 10.2307/1443171

[BIO060150C54] Parmesan, C. (2006). Ecological and evolutionary responses to recent climate change. *Ann. Rev. Ecol. Evol. Syst.* 37, 637-669. 10.1146/annurev.ecolsys.37.091305.110100

[BIO060150C55] Peig, J. and Green, A. J. (2009). New perspectives for estimating body condition from mass/length data: The scaled mass index as an alternative method. *Oikos* 118, 1883-1891. 10.1111/j.1600-0706.2009.17643.x

[BIO060150C56] Pianka, E. (1986). *Ecology and Natural History of Desert Lizards: Analyses of the Ecological Niche and Community Structure*. New Jersey, USA: Princeton University Press.

[BIO060150C57] Qu, Y., Li, H., Gao, J., Xu, X. and Ji, X. (2011). Thermal preference, thermal tolerance and the thermal dependence of digestive performance in two *Phrynocephalus* lizards (Agamidae), with a review of species studied. *Curr. Zool.* 57, 684-700. 10.1093/czoolo/57.6.684

[BIO060150C58] R Core Team (2020). R: A language and environment for statistical computing. R Foundation for Statistical Computing.

[BIO060150C59] Ramesh, M. and Ishwar, N. (2008). *Status and distribution of the Indian Spiny-tailed Lizard Uromastyx hardwickii in the Thar desert, Western Rajasthan*. Technical Report.

[BIO060150C60] Sala, O. E., Stuart Chapin, F., Armesto, J. J., Berlow, E., Bloomfield, J., Dirzo, R., Huber-Sanwald, E., Huenneke, L. F., Jackson, R. B., Kinzig, A. et al. (2000). Global biodiversity scenarios for the year 2100. *Science* 287, 1770-1774. 10.1126/science.287.5459.1770 10710299

[BIO060150C61] Sinervo, B., Méndez-de-la-Cruz, F., Miles, D. B., Heulin, B., Bastiaans, E., Villagrán-Santa Cruz, M., Lara-Resendiz, R., Martínez-Méndez, N., Calderón-Espinosa, M. L., Meza-Lázaro, R. N. et al. (2010). Erosion of lizard diversity by climate change and altered thermal niches. *Science* 328, 894-899. 10.1126/science.1184695 20466932

[BIO060150C62] Stuart-Fox, D., Newton, E. and Clusella-Trullas, S. (2017). Thermal consequences of colour and near-infrared reflectance. *Philos. Trans. R. Soc. B Biol. Sci.* 372, 20160345. 10.1098/rstb.2016.0345 PMC5444066 28533462

[BIO060150C63] Sunday, J. M., Bates, A. E., Kearney, M. R., Colwell, R. K., Dulvy, N. K., Longino, J. T. and Huey, R. B. (2014). Thermal-safety margins and the necessity of thermoregulatory behavior across latitude and elevation. *Proc. Natl Acad. Sci. USA* 111, 5610-5615. 10.1073/pnas.1316145111 24616528 PMC3992687

[BIO060150C64] Vyas, R., Mohapatra, P. and Papenfuss, T. (2022). *Saara hardwickii* (amended version of 2021 assessment). *The IUCN Red List of Threatened Species* 2022, e.T199817A216173870. 10.2305/IUCN.UK.2022-1.RLTS.T199817A216173870.en Accessed on 07 October 2022.

[BIO060150C65] Wilms, T., Wagner, P., Shobrak, M., Rödder, D. and Böhme, W. (2011). Living on the edge? – On the thermobiology and activity pattern of the large herbivorous desert lizard *Uromastyx aegyptia microlepis* Blanford, 1875 at Mahazat as-Sayd Protected Area, Saudi Arabia. *J. Arid Environ.* 75, 636-647. 10.1016/j.jaridenv.2011.02.003

[BIO060150C66] Wood, S. and Wood, M. S. (2015). Package ‘mgcv’. *R Package. version* 1–7.

