# Supplementary material for: Hotter deserts and the impending challenges for the Spiny-tailed Lizard in India
Source: Biol Open. 2024 Mar 2;13(4):bio060150. doi: 10.1242/bio.060150 (PMC11007731; doi:10.1242/bio.060150)
Supplement: Supplementary information [file biolopen-13-060150-s1.pdf]

a)

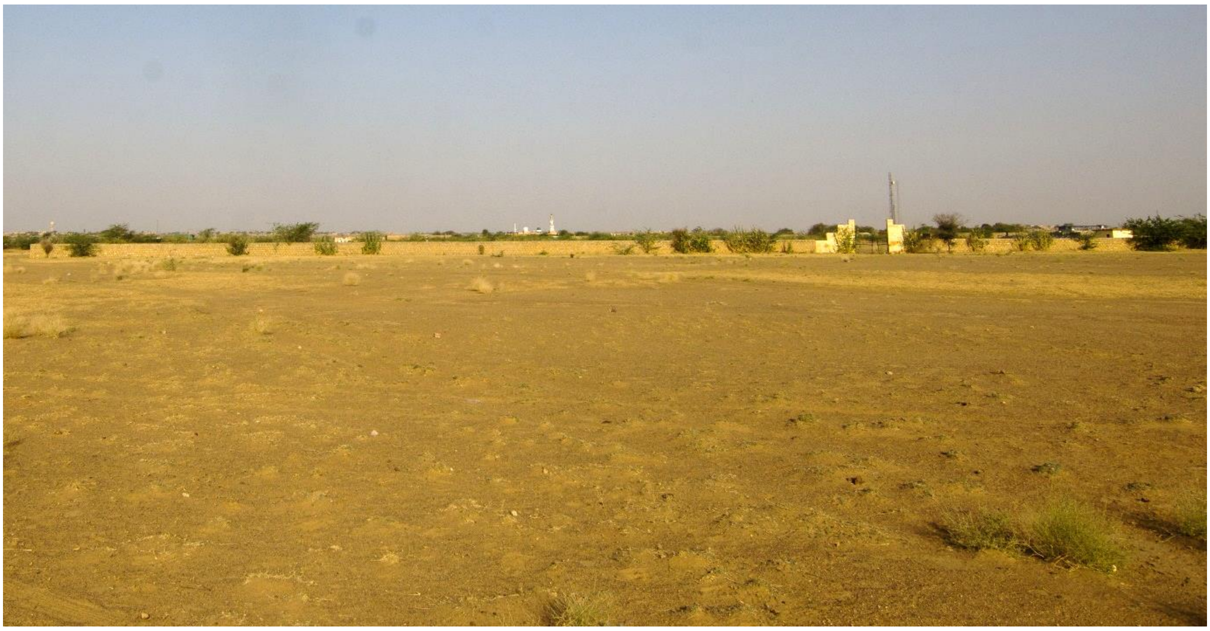

b)

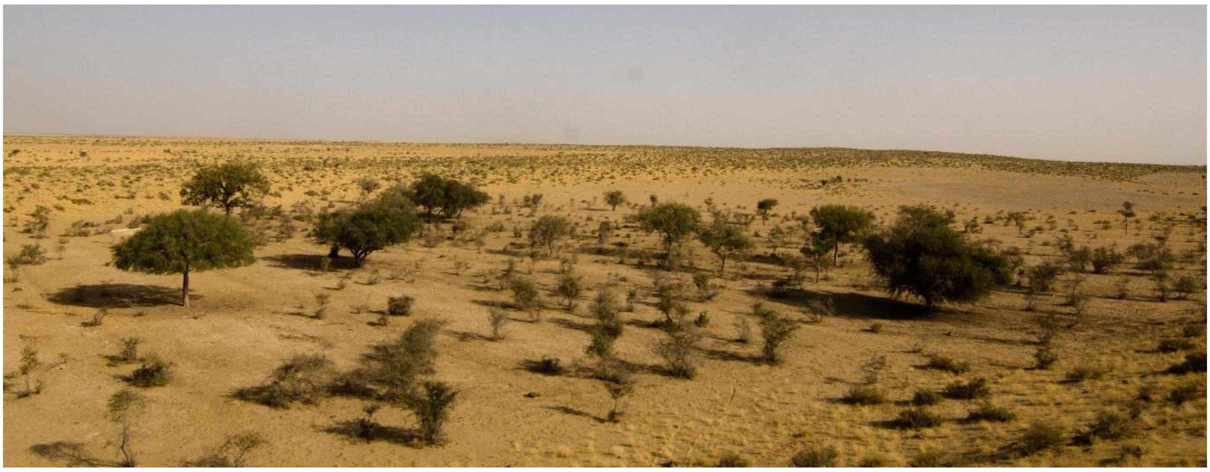

Fig. S1. Photos of the field sites – a) Sam (26°49'32.5"N 70° 30' 42.3"E) b) Bedhiya (26°52'07.3"N 70°27'36.6"E)

Biology Open • Supplementary information

Supporting Information S2

Objective: To investigate differences in operative temperatures of copper model that differ in sizes to emulate sub-adult and adult lizards

Methods: We created copper models that emulate adult (400×35 mm) and sub-adult (250×25 mm) lizards of *S. hardwickii*. Models were molded from copper sheets to be hollow and the length included the tail. Temperature loggers (Maxim Thermochron iButton DS1923; accuracy ±0.5°C) were positioned in the middle of the copper models using a plastic ring and were set to record operative temperature every 15 minutes. We placed these copper models (n=2 of each size) in the open habitat for 47 days and in burrows for 21 days at the Sam site 30 cm apart from each other.

Results: Copper models of adult and juvenile lizards did not show significant differences in operative temperature in open habitats ($F(1,32159) = 0.54$, $P = 0.42$) or in the burrow ($F(1,9742) = 0.31$, $P = 0.08$). Thus, for the estimation of Te across sites and microhabitats, we used only the adult size.

Biology Open • Supplementary information

Supporting Information S3

Objective: To calibrate copper model temperatures and skin temperatures with cloacal temperatures.

Methods: Wild lizards of different size classes (N = 3 total) were caught and housed together in a terrarium at the field station. To determine the degree to which copper models (Te) correlate with skin temperature and cloacal temperature, we placed one adult sized copper model and one lizard in a metal box within the testing terraria. Skin temperatures were measured using the thermal logger in the harness attached to the skin of the lizard (Fig. S2) and cloacal temperatures (core temperatures) were measured using a k-type thermocouple, connected to an external temperature logger (Amprobe model TMD-50).

Over the course of 5 hours on 3 different days, testing terrarium temperatures were steadily increased using a 150W infrared lamp up to 46°C, or steadily decreased using icepacks until 18°C. Cloacal temperatures, skin temperature, and copper model temperatures were recorded every five minutes.

Result: We found a strong positive correlation between skin temperature and cloacal temperature (R^2 = 0.99, $F(1,70)$ = 6273, P<0.001), with 90% of the paired measurements being within ±1°C of each other. There was no significant difference in skin temperature ($F(1,70)$ = 1.42, P = 0.23) or cloacal temperature ($F(1,70)$ = 1.91, P = 0.17) between the three sizes of lizards. Copper model temperatures were also correlated with both skin (R^2 = 0.98, $F(1,70)$ = 5791.2, P<0.001), and cloacal temperature (R^2 = 0.99, $F(1,70)$ = 104573, P<0.001). Thus, operative temperatures for the field were calibrated by adding a correction factor derived from a linear model comparing them with cloacal temperatures. The slope of the linear model was 1 and the intercept was 1.78. Hence the equation x-1.78 was considered as the correction factor for all copper model temperatures. (Fig. S3)

Biology Open • Supplementary information

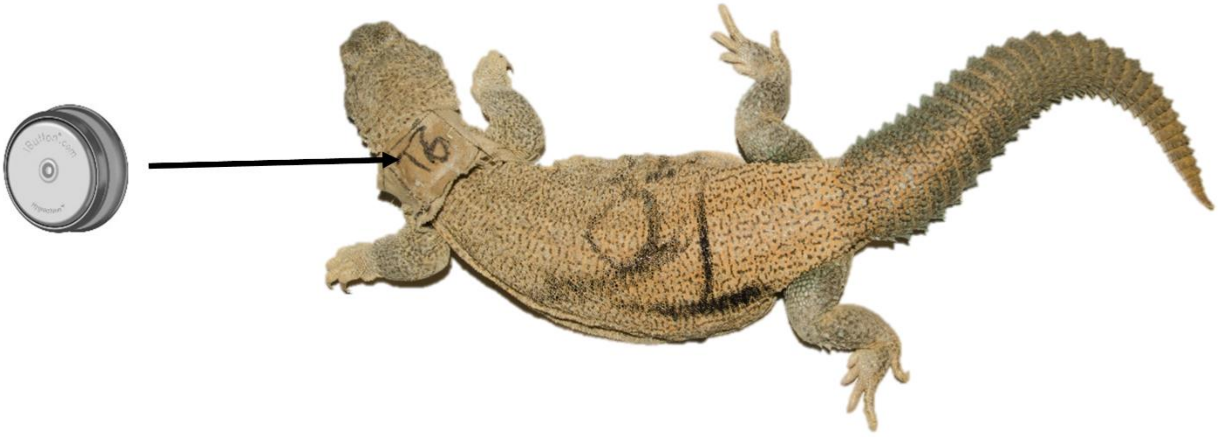

Fig. S2. Image of the harness with Maxim Thermochron iButton DS1921G attached to the lizard

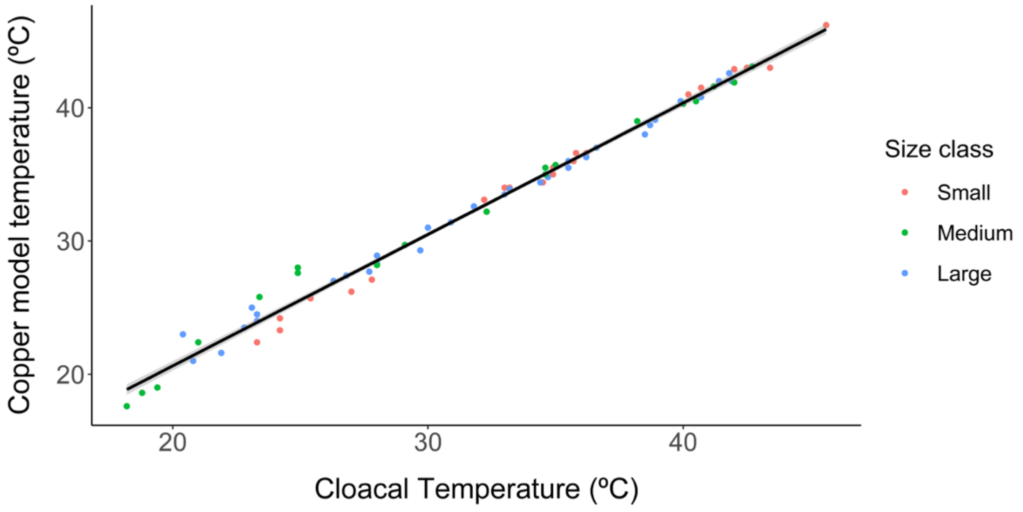

Fig. S3. Copper model temperatures are strongly correlated with cloacal temperatures of *Saara hardwickii*. A correction factor of x-1.78 was derived from this model.

Supporting Information S4

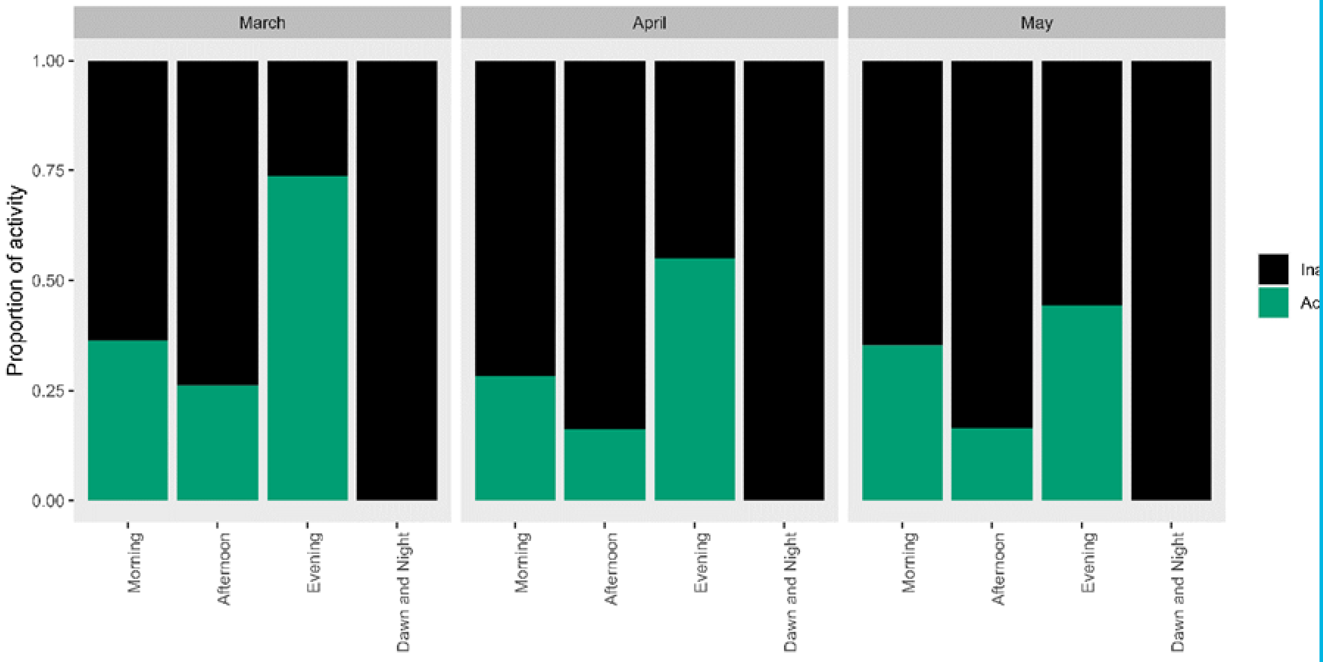

Fig. S4. Average daily activity pattern of the lizards over the course of their active season in the periods designated as Morning (0700 – 1200), Afternoon (1200 – 1700), Evening (1700 – 2000), and Dawn and Night (2000 to 0700). Shown are the proportion of lizards that were active (outside their burrow – in green) and inactive (inside their burrow – in black) at different parts of the day (n = 19 lizards).

Biology Open • Supplementary information

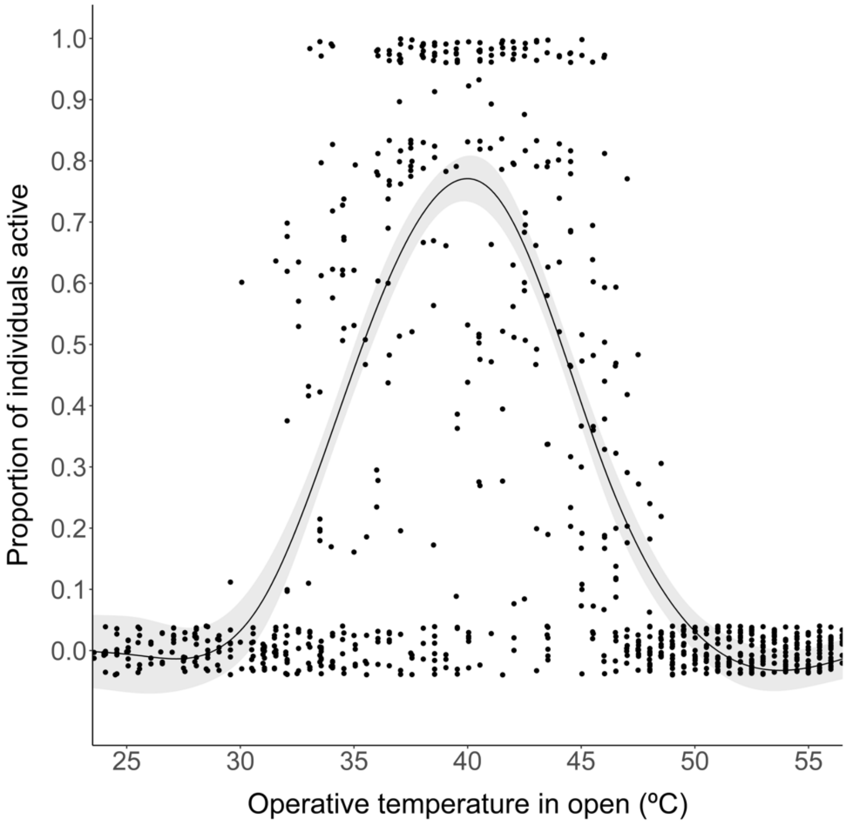

Fig. S5. Temperature-dependence of activity in *Saara hardwickii* (measured as the proportion of individuals recorded outside burrows). Proportion of individuals were derived from field scans (N = 1127 scans) conducted from 0700-1900 hrs, during which operative temperatures in open were also measured. Shown is a smoothing line from Generalised Additive Model.

Biology Open • Supplementary information
